# Human mitochondrial helicase Twinkle has RNA binding, annealing, and strand-exchange activities

**DOI:** 10.1093/nar/gkag008

**Published:** 2026-01-21

**Authors:** Anupam Singh, Laura C Johnson, Noe Baruch-Torres, Y Whitney Yin, Smita S Patel

**Affiliations:** Department of Biochemistry and Molecular Biology, Robert Wood Johnson Medical School, Rutgers University, Piscataway, NJ 08854, United States; Department of Biochemistry and Molecular Biology, Robert Wood Johnson Medical School, Rutgers University, Piscataway, NJ 08854, United States; Graduate Program in Biochemistry, Rutgers University, Piscataway, NJ 08854, United States; Department of Biochemistry and Molecular Biology, University of Texas Medical Branch, Galveston, TX 77555, United States; Sealy Center for Structural Biology, University of Texas Medical Branch, Galveston, TX 77555, United States; Department of Biochemistry and Molecular Biology, University of Texas Medical Branch, Galveston, TX 77555, United States; Sealy Center for Structural Biology, University of Texas Medical Branch, Galveston, TX 77555, United States; Department of Biochemistry and Molecular Biology, Robert Wood Johnson Medical School, Rutgers University, Piscataway, NJ 08854, United States; Graduate Program in Biochemistry, Rutgers University, Piscataway, NJ 08854, United States

## Abstract

Twinkle is the sole replicative helicase in human mitochondria, essential for mitochondrial DNA replication. Beyond its canonical unwinding activity, Twinkle has non-canonical activities, including DNA annealing and strand-exchange. Here, we show that these non-canonical activities extend to RNA. Twinkle binds RNA and catalyzes RNA:DNA hybrid formation through annealing, strand-exchange, and toehold-mediated strand displacement. Twinkle can unwind RNA:DNA forks when loaded onto the DNA tail but not the RNA tail. Although the physiological role of these RNA-related activities remains unclear, we show that Twinkle can strand-exchange an RNA downstream of a stalled replication fork to restart replication. The annealing/strand-exchange activity can be involved in DNA replication initiation and repair, but RNA:DNA hybrids can compromise genome integrity, emphasizing the need to balance unwinding and annealing activities. Interestingly, mitochondrial SSB inhibits the RNA:DNA annealing activity of Twinkle, thus regulating the non-canonical functions of Twinkle. A disease-associated W315L variant, which is defective in DNA replication, retains annealing and strand-exchange functions with both RNA and DNA, resulting in an imbalance between replication and annealing functions that may underlie its pathogenicity. Our findings of Twinkle’s RNA-binding and strand-exchange activities may have a connection to its localization within mitochondrial RNA granules.

## Graphical Abstract

**Graphical Abstract:**
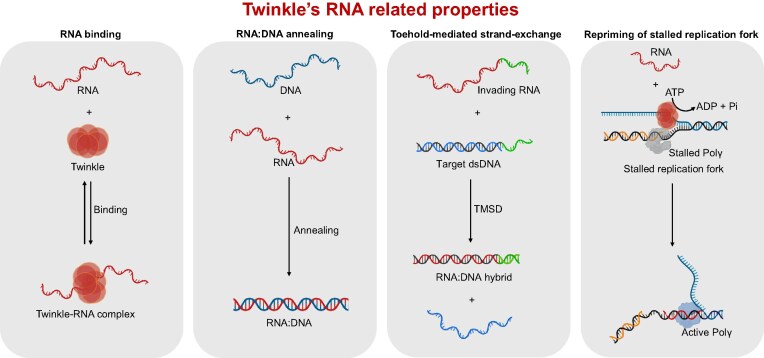

## Introduction

Mitochondria have their own genome, which is maintained in high copy numbers by a replication machinery that is distinct from that of the nuclear genome. The 16.5-kb circular human mitochondrial (mt) DNA is replicated in the mt nucleoids by a core set of enzymes, including DNA polymerase γ (Polγ), mt single-stranded (ss) DNA-binding protein (mtSSB), and the replicative helicase Twinkle [1, 2]. Twinkle is a ring-shaped hexameric helicase, initially identified through its association with the autosomal dominant progressive external ophthalmoplegia (adPEO) gene and its sequence homology to the bacteriophage T7 gp4 helicase-primase [3, 4].

**Figure 1.**
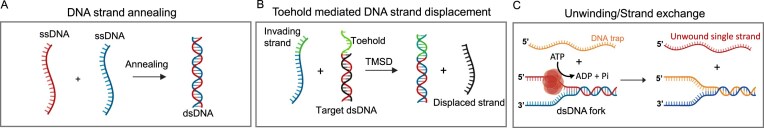
Twinkle’s non-canonical activities. (**A**) Twinkle catalyzes the annealing of two complementary strands of DNA [8]. (**B**) When a short toehold is present in a target dsDNA, Twinkle can mediate strand exchange with a complementary invading strand through a TMSD mechanism, independent of its ATPase activity [9]. (**C**) Twinkle can generate a toehold in a dsDNA fork using its ATPase-dependent unwinding activity and couple this to annealing a complementary DNA strand, linking unwinding to strand-exchange [10].

Unlike T7 gp4, the N-terminal domain of human Twinkle has lost primase activity [5] but retains DNA-binding function, enabling processive strand displacement synthesis by Polγ [6, 7]. Additionally, Twinkle has acquired new capabilities, including DNA annealing and strand-exchange. Twinkle promotes the annealing of complementary DNA strands (Fig. 1A) [8] and mediates strand-exchange between an ssDNA invader and a double-stranded (ds) DNA target via a short (>1 nt) toehold region (Fig. 1B). This toehold-mediated strand displacement (TMSD) occurs independently of its ATPase activity [9]. Twinkle can also exploit its ATPase-driven unwinding to generate a toehold in a dsDNA fork *in situ*, enabling annealing of a complementary ssDNA strand and thereby coupling unwinding to strand-exchange (Fig. 1C) [10].

The physiological role of the non-canonical activities of Twinkle remains unclear, but they may contribute to DNA repair processes and be linked to adPEO, a disorder characterized by the accumulation of multiple mtDNA deletions [11]. Unlike the nucleus, mitochondria lack robust DNA repair and recombination pathways. However, replication proteins, including Twinkle, have been implicated in repair processes [12–17]. Recent evidence suggests that dsDNA breaks in mitochondria are rarely repaired and more often lead to degradation of mtDNA [18, 19]; however, repair may occur when replication stalling or DNA breaks are more frequent, particularly with Twinkle variants that show reduced replication efficiency [20]. Under such conditions, error-prone repair [21] or copy-choice recombination [22] involving misannealing between direct repeats give rise to mtDNA deletions. Thus, Twinkle’s DNA annealing activity could contribute to pathogenic outcomes.

A recent study demonstrated that, in addition to its localization within mt nucleoids, Twinkle is also a prominent component of mt RNA granules, where it contributes to their formation and stability [23]. This observation raises the possibility that Twinkle may carry out RNA-related functions. Both RNA:DNA hybrids [24, 25] and triple-stranded R-loops [26, 27] have been detected in mitochondria, although their origins remain unclear. These RNA:DNA hybrids are thought to play important roles in DNA replication and transcription regulation [26, 28, 29]. Notably, knockdown of Twinkle expression in human cells reduces the abundance of mt RNA:DNA hybrids [23], whereas overexpression of Twinkle in Drosophila increases the amount of RNA associated with replication intermediates [13]. Given that Twinkle has DNA strand annealing activity, we asked whether it might also catalyze the formation of RNA:DNA hybrids, a possibility suggested previously [28], but not experimentally tested.

To explore the role of Twinkle in RNA metabolism, we biochemically investigated its RNA-binding and RNA-related activities using purified proteins. We found that Twinkle binds RNA with high affinity and catalyzes RNA:DNA annealing as well as RNA-driven strand exchange with dsDNA. Moreover, Twinkle can couple these activities to restart DNA synthesis at a stalled replication fork by annealing an RNA primer. We show that a disease-associated Twinkle variant, while defective in replication, retains near-normal strand-exchange activity, suggesting that an imbalance between replication and annealing/strand-exchange functions may contribute to mt genome instability.

## Materials and methods

### Oligonucleotides

DNA and RNA oligonucleotides were ordered from Integrated DNA Technology (IDT) or TriLink BioTechnologies (Supplementary Table S1). “70-nt RNA” and “40-nt RNA Trap” were synthesized using *in vitro* transcription as described previously [30].

### Proteins

Twinkle (amino acids 42–684) and Twinkle variant W315L were cloned in pET28 SUMO expression vector and expressed in *Escherichia coli* Rosetta (DE3) cells with 0.5 mM isopropyl β-D-thiogalactopyranoside (IPTG). Bacterial cell pellet with expressed protein was resuspended in a buffer consisting of 20 mM HEPES (pH 8.0), 1 M NaCl, 1 mM phenylmethylsulfonyl fluoride (PMSF), 10% glycerol, 0.1% Triton X-100, 5 mM β-mercaptoethanol, 0.2 mg.ml^−1^ lysozyme and Roche protease inhibitor tablets, and lysed by sonication. Cell lysate was centrifuged at 30,000 *g* for 45 min at 4°C and the supernatant was incubated with Qiagen Ni-NTA agarose resin for ~1 h. Resin was washed with lysis buffer supplemented with 50 mM imidazole and 2 M NaCl followed by washes with buffers containing 700 and 400 mM NaCl. The protein was eluted with lysis buffer containing 400 mM NaCl and 300 mM imidazole. The sample was diluted to 300 mM NaCl with buffer A [20 mM HEPES, pH 8.0, 10% glycerol, 2 mM EDTA (ethylenediaminetetraacetic acid), pH 8.0, 5 mM β-mercaptoethanol] and loaded to a HiTrap heparin HP column (Cytiva). Bound SUMO-Twinkle was eluted with a continuous NaCl gradient (300–1000 mM). Fractions with pure SUMO-Twinkle protein were pooled and SUMO-tag was cleaved with ULP1 SUMO-protease treatment while dialyzing the protein overnight in buffer A supplemented with 500 mM NaCl and 1 mM dithiothreitol (DTT). Protein was further purified, and SUMO tag was removed using a Superdex 200 gel filtration column. After gel filtration, pure fractions were pooled and concentrated in buffer A containing 700 mM NaCl and stored at −80°C.

PolγA, PolγB, and mtSSB were purified as described previously [7].

### Nucleic acid binding affinity by fluorescence anisotropy-based titrations

The equilibrium dissociation constant (*K*_D_) of FAM-labeled DNA or RNA complexes with Twinkle was measured using fluorescence anisotropy-based titrations. All binding reactions were conducted at 25°C, and fluorescence anisotropy was measured on a plate-based fluorometer (Tecan Spark, Switzerland). Briefly, 2.5 nM of DNA or RNA was incubated with a range of Twinkle concentrations (T) in reaction buffer (50 mM Tris-acetate, pH 7.5, 0.01% tween 20, 1 mM EDTA, and 5 mM DTT). Equilibrium binding was detected by measuring fluorescence anisotropy (*p*) (excitation wavelength = 485 nm, emission wavelength = 535 nm). The titration data were fit to eq. (1) to determine the *K*_D_ values.


(1) \begin{eqnarray*}
A = \frac{{{{A}_{\mathrm{ max}}}*{{{\left[ T \right]}}^h}}}{{\left( {{{K}_{\mathrm{D}}} + {{{\left[ T \right]}}^h}} \right)}} + c.
\end{eqnarray*}

Here, *A* is fluorescence anisotropy at Twinkle concentration [T], *A*_max_ is the maximum anisotropy when all the DNA is bound to Twinkle, *c* is the anisotropy of free DNA or RNA, *K*_D_ is the dissociation constant of the Twinkle–nucleic acid complex, and *h* is the Hill coefficient. A similar strategy was used to measure equilibrium binding of mtSSB to ssDNA and RNA.

**Figure 2.**
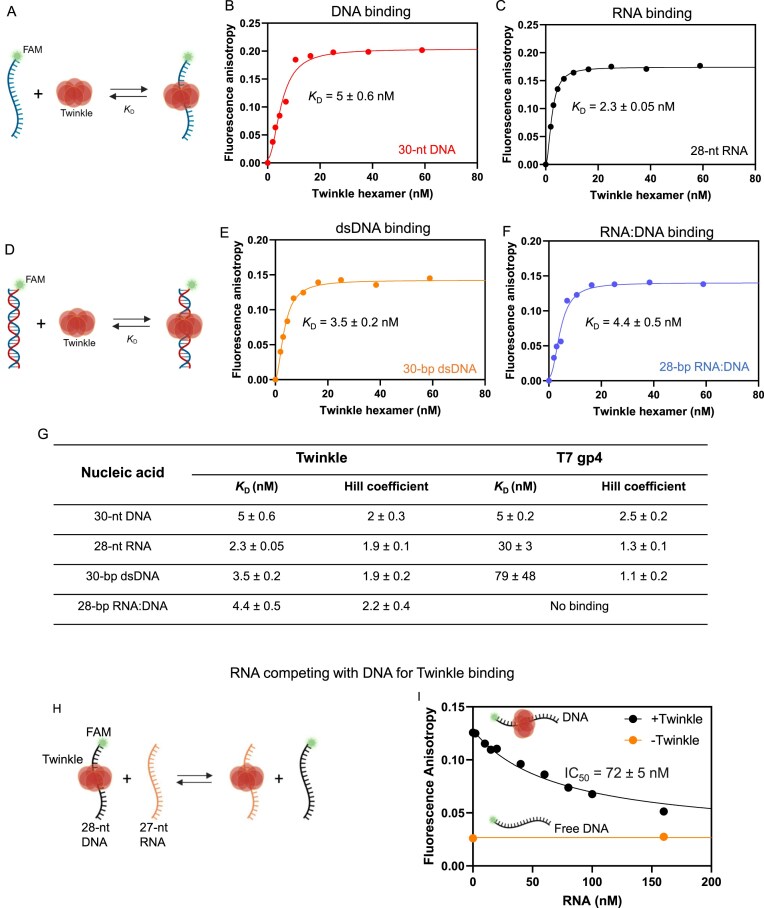
Twinkle binds to RNA and RNA:DNA hybrid substrates. (**A**) Twinkle binds to FAM-labeled DNA or RNA. Fluorescence anisotropy-based titrations measure Twinkle binding to 2.5 nM FAM-labeled 30-nt ssDNA (**B**) or FAM-labeled 28-nt RNA (**C**). Data represent the mean from *N* = 2 (Supplementary Fig. S1 and Supplementary Table S1). Lines are fit to eq. (1); *K*_D_ values are shown in panel (G). (**D**) Twinkle binds to FAM-labeled dsDNA and RNA:DNA. Anisotropy titrations with 2.5 nM FAM-labeled dsDNA (**E**) or RNA:DNA hybrid (**F**), mean *N* = 2; solid lines are fits to eq. (1). (**G**) Summary of *K*_D_ and Hill coefficients (±SE from fitting) from panels (B), (C), (E), and (F), and Supplementary Fig. S1C and D. (**H**) RNA competes with DNA for binding to Twinkle. (**I**) Fluorescence anisotropy of Twinkle-bound 28-nt ssDNA decreases with increasing unlabeled 27-nt RNA (black circles, mean *N* = 2). Orange circles show free ssDNA and ssDNA with 160 nM RNA. Data were fit to eq. (2) to determine the IC_50_ value.

For the competition experiment (Fig. 2H and I), 40 nM FAM-labeled ssDNA and stated concentrations of unlabeled RNA were mixed. Forty nanomolars of Twinkle hexamer was added to each reaction and incubated for 20 min at 25°C. Fluorescence anisotropies were measured as stated above and the data were fit to eq. (2) to determine IC_50_.


(2) \begin{eqnarray*}
A = A_0 + \frac{{{{A}_{\mathrm{ max}}} - {{A}_0}}}{{\left( {\frac{{\left[ {RNA} \right]}}{{IC50}}} \right) + 1}}.
\end{eqnarray*}

Here, *A* is fluorescence anisotropy at RNA concentration [RNA], *A_0_* is fluorescence anisotropy of 40 nM FAM-ssDNA and 160 nM RNA in the absence of Twinkle, and *A*_max_ is the maximum anisotropy when all the FAM-ssDNA is bound to Twinkle in the absence of RNA.

Inhibition constant (*K*_i_) was estimated using Cheng and Prusoff equation [31],


(3) \begin{eqnarray*}
Ki = \frac{{IC50}}{{\left( {\frac{{\left[ {\mathrm{ DNA}} \right]}}{{{{K}_{\mathrm{D}}}}}} \right) + 1}},
\end{eqnarray*}

where [DNA] is the DNA concentration used (40 nM) and *K*_D_ is the dissociation constant of RNA–Twinkle complex (2.3 nM).

### Stopped-flow method to measure strand annealing kinetics

The kinetics of nucleic acid annealing were measured using a stopped-flow device (Kintek Corp.) connected to a fluorescence detector. A 20 nM solution of 3′ FAM-labeled DNA or RNA was added to syringe A, and 80–1200 nM of the complementary DNA strand with quenching dG residues at its 5′-end to syringe B. The solutions were prepared in a buffer containing 50 mM Tris-acetate (pH 7.5), 0.01% tween 20, 1 mM EDTA, 50 mM sodium acetate, and 2 mM DTT. In Twinkle-catalyzed annealing reactions, 80 nM Twinkle hexamer was added in syringe A with the FAM-labeled RNA or ssDNA. Annealing experiments with mtSSB were carried out by incubating mtSSB (10–50 nM tetramer) with the complementary strand. Equal volumes of solutions A and B were rapidly mixed, and FAM-fluorescence was measured in real time from milliseconds to minutes time range. For the strand annealing reactions with very slow kinetics, solutions A and B were rapidly mixed manually, and fluorescence intensity was measured on a plate-based fluorometer (Tecan Spark, Switzerland). Reactions were performed at 25°C. Traces from spontaneous kinetic annealing reactions were fit to a single-exponential equation: 


(4) \begin{eqnarray*}
Y = A*{{e}^{ - kt}} + {{Y}_0}.
\end{eqnarray*}

*Y* is fluorescence intensity at time “*t*,” *Y*_0_ is the initial fluorescence, *A* is the amplitude, and *k* is the observed rate.

Fluorescence traces from Twinkle-catalyzed annealing reactions were fit to a two-phase exponential equation:


(5) \begin{eqnarray*}
Y = A1*{{e}^{ - k1t}} + A2*{{e}^{ - k2t}} + {{Y}_0}.
\end{eqnarray*}

A weighted average of *k*_1_ and *k*_2_ was used to determine the observed rate of annealing.

### TMSD kinetics

Observed rates of TMSD reactions were measured on a stopped-flow device. FAM-labeled invader DNA or RNA was incubated with Twinkle in syringe A, and the target dsDNA was filled in syringe B. The solutions were rapidly mixed, and FAM fluorescence was measured in real time. To measure spontaneous TMSD rate, reactions were performed in the absence of Twinkle. The final reaction consisted of 10 nM target dsDNA, 40 nM RNA or DNA invader, and 40 nM wild-type or W315L Twinkle. TMSD experiments with mtSSB were carried out by incubating mtSSB (10–40 nM tetramer) with the invader strand. Reaction buffer consisted of 50 mM Tris-acetate (pH 7.5), 10% glycerol, 2 mM DTT, 0.01% tween 20, 1 mM EDTA and 50 mM sodium acetate. All the reactions were performed at 25°C. Fluorescence traces were fit to the single-exponential eq. (4).

### Fork unwinding reactions

Short DNA:DNA and two different RNA:DNA forks with 40 bp double-stranded region and 25–30-nucleotide-long 5′ and 3′ tails were annealed by heating to 95°C and slow cooling to room temperature. The theoretical melting temperatures of the forks are as follows: DNA:DNA fork: 72.7°C; DNA:RNA fork (5′ DNA tail): 75.3°C; RNA:DNA fork (5′ RNA tail): 72.1°C (IDT OligoAnalyzer™, https://www.idtdna.com/pages/tools/oligoanalyzer).

To measure the unwinding activity, 10 nM fork substrate was incubated with 55.5 nM Twinkle for 20 min in the unwinding buffer containing 50 mM Tris-acetate (pH 7.5), 0.01% tween 20, 5 mM DTT, and 1 mM EDTA. The unwinding reaction was initiated by adding 8 mM magnesium acetate, 4.5 mM ATP, and 100 nM unlabeled trap DNA with the same sequence as the strand that forms the 5′ tail of the fork substrate or 10 nM mtSSB (tetramer concentration). Control reactions lacking either the trap DNA or ATP were similarly conducted. Reactions were quenched at the time points noted in the figures with the addition of 50 mM EDTA and 0.5% sodium dodecyl sulfate (SDS), and the reaction products were resolved on a 4%–20% TGX gel. The gels were scanned using the GE Typhoon 950 imaging system. The fractions of the product ssDNA and ds fork substrate for each time-point were quantified from the gel using the ImageQuant package. The product fractions were plotted as a function of time and were fit to the single-exponential eq. (4) to obtain the kinetics of strand-exchange. The initial rate of strand-exchange was determined as


(6) \begin{eqnarray*}
\mathrm{ Initial }\ \mathrm{ rate} = A*k,
\end{eqnarray*}

where *A* and *k* are the amplitude and observed rate, respectively, determined from exponential fitting of data.

### ATPase activity

The ATPase activity of Twinkle in absence or in presence of 70-nt ssDNA, 70-nt RNA (see “3′-tail strand” and “Trap DNA” sequences of the DNA:RNA fork with DNA 5′-tail in Supplementary Table S1), DNA:DNA fork, RNA:DNA fork, or DNA:RNA fork substrates was determined using NADH-coupled ATPase assay. Briefly, as Twinkle hydrolyzes ATP to ADP, a coupled enzyme system consisting of pyruvate kinase and lactate dehydrogenase regenerates ATP and consumes NADH [32]. The kinetics of NADH decrease was determined by measuring NADH fluorescence (excitation wavelength = 335 nm, emission wavelength = 440 nm). Reactions contained 0.2 mM NADH, 1 mM phosphoenolpyruvate, 30 U/ml pyruvate kinase, 30 U/ml lactate dehydrogenase, 5 mM magnesium chloride, 60 nM Twinkle hexamer, and 100 nM nucleic acid substrates when present. Reactions were initiated with the addition of 2 mM ATP and fluorescence was measured over time on a plate-based fluorometer (Tecan Spark, Switzerland). The rate of ATP hydrolysis was determined using an NADH standard curve. All the reactions were performed at 25°C.

### DNA replication assays

To test strand displacement synthesis by Polγ in the presence of wild-type or W315L mutant Twinkle, a forked DNA substrate with a 45-nt 5′-tail, a 34-nt 3′-tail, and a 40 bp ds region was used. A 30-nt 5′-FAM-labeled primer was annealed to the 3′-tail of the fork, leaving a gap of 4-nt between the 3′-primer-end and fork junction. Reactions were carried out in buffer containing 20 mM HEPES (pH 8.0), 105 mM KCl, 35 mM NaCl, 1 mM DTT, 2% glycerol, and 0.1 mg.ml^−1^ bovine serum albumin . A 100 nM primed forked DNA substrate, 200 nM Polγ, and 300 nM of either wild-type Twinkle or the W315L mutant were combined and incubated at 37°C for 5 min. To initiate the reaction, a mixture of 10 mM MgCl_2_, 0.2 mM dNTPs, and 4 mM ATP was added. Reactions were quenched after 10 min with 80% formamide, 50 mM EDTA (pH 8.0), 0.1% SDS, 5% glycerol, and 0.02% bromophenol blue, boiled at 95°C for 5 min, and resolved on a 17% polyacrylamide gel containing 8 M urea. The bands were visualized using FAM-fluorescence on a GE Typhoon FLA 9000 Gel Scanner (GE Healthcare), and the products were quantified using ImageQuant TL (GE Healthcare). The percentage of full-length product was calculated using the ratio of full-length extension band intensity to the sum of gap-filling product intensities.

Replication restart reactions on a stalled replication fork (with a ddCMP-terminated primer) were performed by assembling 50 nM forked DNA with 200 nM Polγ and 300 nM Twinkle hexamer in a buffer containing 50 mM Tris–HCl (pH 7.5), 10% glycerol, 0.01% tween 20, and 1 mM EDTA. Strand-exchange and DNA synthesis reactions were initiated by the addition of 50 nM 20-nt 5′-FAM-labeled RNA, 2 mM ATP, 10 mM MgCl_2_, and 100 µM dNTPs. When mtSSB was present, it was added with the RNA trap. Reactions were quenched at the mentioned time-points by the addition of 83 mM EDTA. Quenched reactions were heated at 95°C with 6 µM unlabeled upper strand to inhibit reannealing of the RNA trap with the template strand during the gel run. Reaction products were resolved on a 15% urea-TBE gel, and the gel was scanned for FAM fluorescence. The percentage of extended RNA was then quantified.

### Statistical analysis

Data fitting of the stopped-flow kinetic traces was performed using KinTek Explorer software [33, 34]. All other curve-fitting and error analyses were performed using GraphPad Prism software. At least two independent repeats were performed for all experiments, and bar charts with independent data points are presented. Standard errors of means are shown with the bar charts for the experiments with three or more repeats.

## Results

### Twinkle binds RNA and RNA:DNA hybrid

To assess the binding affinity of Twinkle for RNA and DNA substrates, we used a fluorescence anisotropy-based assay with fluorescein-labeled nucleic acid substrates, including RNA, RNA:DNA, ssDNA, and dsDNA. A constant amount of the nucleic acid was titrated with increasing amounts of Twinkle, and the resulting binding data were fit to the Hill equation [eq. (1)] to obtain the nucleic acid–Twinkle complex *K*_D_ values (Fig. 2A–G). Consistent with previous reports [7–9, 35], Twinkle showed a strong binding affinity for both ssDNA (Fig. 2B) and dsDNA (Fig. 2E), with a measured *K*_D_ of ~3–5 nM. Interestingly, Twinkle also showed a similarly high affinity for the RNA (Fig. 2C) and RNA:DNA hybrid (Fig. 2F and G, and Supplementary Fig. S1A).

We were intrigued that Twinkle, a DNA replicative helicase, interacts strongly with RNA. To determine whether this property is unique to Twinkle, we examined the RNA-binding ability of its phage homolog, the T7 gp4 helicase. Previous studies have shown that T7 gp4 requires dTTP or its analogs to bind to ssDNA [36, 37]. Therefore, nucleic acid binding studies with T7 gp4 were conducted in the presence of the non-hydrolyzable nucleotide analog dTMPPCP. Under these conditions, T7 gp4 bound ssDNA with high affinity (*K*_D_ of 5 nM), which is comparable to Twinkle, but its RNA binding affinity was 13-fold weaker (*K*_D_ of 30 nM) than Twinkle (Fig. 2G and Supplementary Fig. S1B and C). T7 gp4 also showed a weak affinity for dsDNA (*K*_D_ of 79 nM) and almost no binding to the RNA:DNA hybrid (Fig. 2G and Supplementary Fig. S1B and D).

To determine whether RNA competes with DNA for binding to Twinkle, we designed a competition assay in which a complex of 40 nM Twinkle hexamer with a 40 nM FAM-labeled 28-nt ssDNA was titrated with an increasing concentration of unlabeled 27-nt RNA. The competition data showed that RNA fully displaced the ssDNA bound to Twinkle with an IC_50_ of 72 nM (Fig. 2H and I, and Supplementary Fig. S1E). A competitive model [eq. (3)] provided an inhibition constant (*K*_i_) of 8 nM, which is close to the experimentally determined *K*_D_ for RNA binding to Twinkle (2.3 nM). This suggests that RNA binds to Twinkle at the same site as the DNA. However, it is also possible that Twinkle has multiple nucleic acid binding sites.

This study provides the first evidence that Twinkle binds RNA and RNA:DNA hybrids with a high affinity, linking this property to recent observations of Twinkle in the mtRNA granules [23]. Whether Twinkle’s RNA-binding activity represents an evolutionary adaptation for specialized mt functions beyond its established role in DNA replication remains to be determined.

### Twinkle unwinds RNA:DNA hybrid

**Figure 3.**
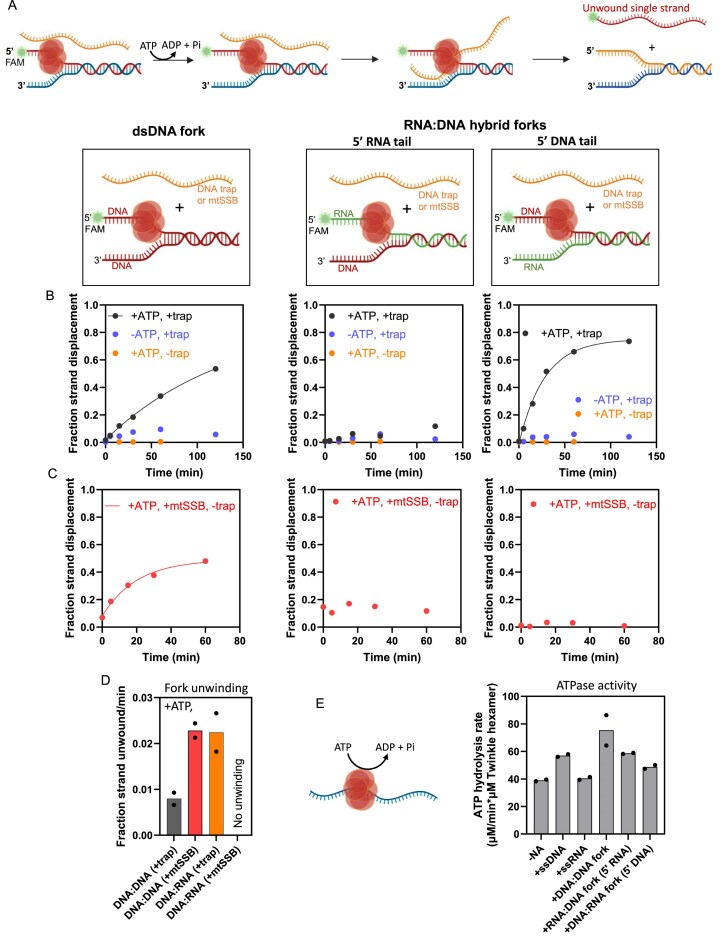
Twinkle resolves RNA:DNA hybrid fork. (**A**) Schematic of the trap-dependent unwinding/strand-exchange mechanism catalyzed by Twinkle. (**B**) Unwinding of DNA:DNA and RNA:DNA forks (with 5′-RNA or 5′-DNA tails) by Twinkle. Reactions contained 10 nM fork substrate, 55.5 nM Twinkle hexamer, and 100 nM complementary DNA trap at 25°C. The FAM-labeled unwound strand was resolved by native polyacrylamide gel electrophoresis (PAGE) (Supplementary Fig. S2). The Fraction of unwound product is plotted over time with controls lacking ATP or a DNA trap. Data are representative of two independent experiments; solid lines are fits to a single-exponential function [eq. (4)]. (**C**) Unwinding of DNA:DNA and DNA:RNA hybrid forks by Twinkle in the presence of mtSSB without DNA trap. Data are representative of two independent experiments; lines are fits to eq. (4). (**D**) Bar chart summarizing unwinding/strand-exchange rates of Twinkle under the conditions shown in panels (B) and (C). (**E**) ATPase activity of Twinkle in the absence or presence of ssDNA, RNA, DNA:DNA, or RNA:DNA hybrid forks (mean *N* = 2).

Given Twinkle’s ability to bind RNA and RNA:DNA, we investigated whether Twinkle unwinds RNA:DNA hybrids to resolve such structures found in the mitochondria. Twinkle requires a preformed forked DNA substrate with 5′- and 3′-ssDNA tails to load and unwind the duplex DNA. Twinkle loads onto the 5′-tail of the fork DNA and uses its ATPase activity to translocate along it and unwind the downstream dsDNA [6, 8, 38]. Twinkle is not processive and, on its own, cannot unwind the 40-bp duplex fork used here. Hence, DNA unwinding was measured in the presence of a DNA trap complementary to the 3′-displaced strand added at the start of the unwinding reaction with MgATP (Fig. 3A).

Under these conditions, Twinkle unwound the 40-bp DNA fork with a 30-nt 5′-ssDNA tail at an initial rate of 0.008 strand fraction·min^−1^ (Fig. 3B, left panel, and D, and Supplementary Fig. S2A and B). By 120 min, ~50% of the DNA was unwound (Fig. 3B, left panel). Both ATP and the DNA trap were required for this activity, and unwinding was minimal in the absence of ATP or the trap (Fig. 3B, left panel, and Supplementary Fig. S2B).

To assess whether Twinkle unwinds RNA:DNA hybrids, two types of hybrid forks were prepared; one contained a 5′-RNA tail (RNA:DNA fork), while the other had a 5′-DNA tail (DNA:RNA fork). Twinkle did not unwind the RNA:DNA fork with a 5′-RNA tail (Fig. 3B, middle panel, and Supplementary Fig. S2C, left panel). However, it successfully unwound the DNA:RNA fork with the 5′-DNA tail with an initial rate of 0.022 strand fraction·min^−1^, ~3× faster than the DNA fork (Fig. 3B, right panel, and D, and Supplementary Fig. S2C, right panel). The faster unwinding of the DNA:RNA fork is not explained by a lower melting temperature, as its melting temperature is similar to that of the DNA:DNA fork (see the “Materials and methods” section). Thus, Twinkle can unwind hybrid fork structures when it loads onto the ssDNA strand, but not when it loads onto the ssRNA strand.

Previous studies have shown that mtSSB facilitates DNA unwinding by Twinkle [38]. To test its role, we measured the unwinding of DNA and RNA:DNA forks in the presence of mtSSB and in the absence of trap DNA. MtSSB supported DNA:DNA fork unwinding with an initial rate of 0.02 strand fraction·min^−1^ (Fig. 3C, left panel, and D, and Supplementary Fig. S2B). In contrast, mtSSB did not support unwinding of the DNA:RNA or the RNA:DNA forks (Fig. 3C, middle and right panels, and Supplementary Fig. S2C), despite its binding RNA with sub-nanomolar affinity (Supplementary Fig. S2D) [23]. If mtSSB acted simply by sequestering the displaced 3′-strand, one would expect it to promote DNA:RNA fork unwinding. Instead, these results suggest that mtSSB plays an active role in Twinkle-mediated unwinding that requires it to bind specifically to the DNA strand.

It is also curious that Twinkle binds to RNA, but it does not promote unwinding of the RNA:DNA fork with a 5′-RNA tail. This defect may stem from its inability to translocate on ssRNA. Because translocation and ATP hydrolysis are coupled, we measured Twinkle’s ATPase activity in the presence of ssRNA. Twinkle has a basal ATPase activity of ~38 min^−1^ in the absence of nucleic acid. This activity increased to ~56 min^−1^ in the presence of a 70-nt ssDNA (Fig. 3E). In contrast, no stimulation of ATPase activity was observed in the presence of a 70-nt RNA (Fig. 3E). The lack of RNA-stimulated ATPase activity indicates that Twinkle cannot translocate on ssRNA, which might be the reason why it does not unwind the RNA:DNA fork substate with a 5′-RNA tail.

We also examined Twinkle’s ATPase activity on each fork substrate (Fig. 3E). Interestingly, the RNA:DNA fork stimulated the ATPase activity, despite not being unwound. We cannot rule out the possibility that this ATPase activity originates from Twinkle molecules bound to the 3′-ssDNA tail.

Overall, our results demonstrate that Twinkle can resolve DNA:RNA hybrids, but it must be translocating on ssDNA. Twinkle does not unwind RNA:DNA forks, where it needs to translocate on ssRNA, and mtSSB does not facilitate unwinding of the hybrid forks.

To ensure that DNA:RNA unwinding activity of Twinkle is dependent on its ATPase activity, we constructed and purified an ATPase-deficient Twinkle mutant E445Q. Based on the reported cryogenic electron microscopy structure of *Lates calcarifer* Twinkle [39], the E445 residue interacts with the γ-phosphate of ATP and Mg^2+^ ion, and the E445Q mutation disrupts this function [40]. The E445Q Twinkle did not unwind the DNA:DNA or the DNA:RNA fork (Supplementary Fig. S3), confirming that the DNA:RNA fork unwinding activity is dependent on the motor functions of Twinkle.

### Twinkle catalyzes DNA:DNA, RNA:DNA, and RNA:RNA annealing reactions

**Figure 4.**
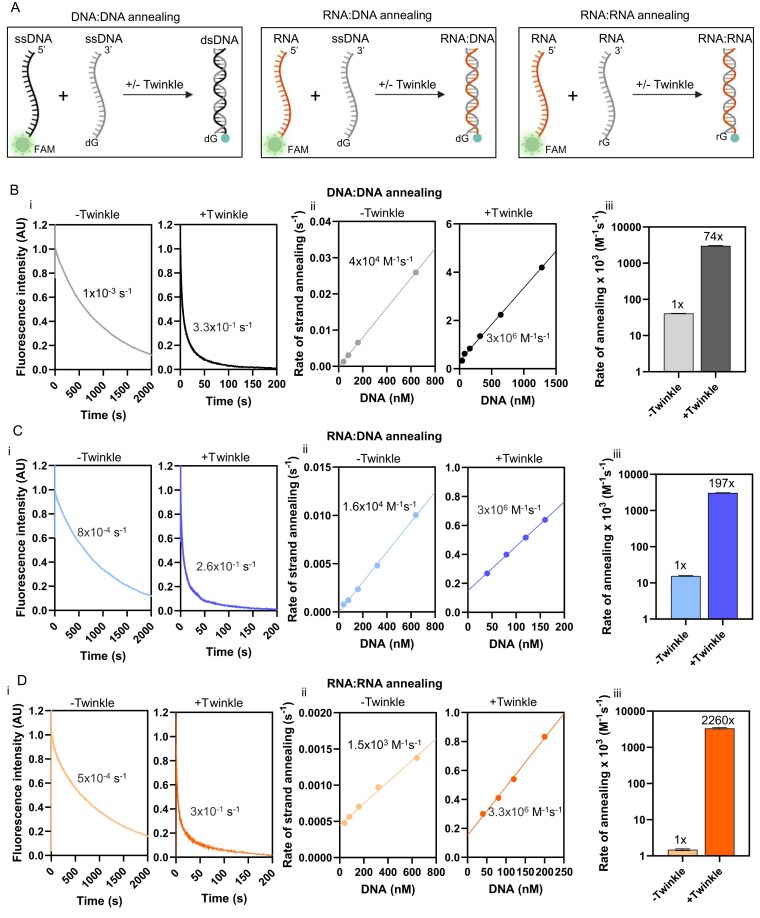
Twinkle has RNA:DNA and RNA:RNA annealing activities. (**A**) Schematic of DNA:DNA (left), RNA:DNA (middle), and RNA:RNA (right) strand annealing. Quenching of FAM fluorescence by dG residues allows real-time monitoring of annealing kinetics. (**B**) DNA:DNA annealing. (i) Representative kinetic traces showing 10 nM FAM-labeled ssDNA annealing to 40 nM unlabeled complementary DNA in the absence or presence of 40 nM Twinkle hexamer. (ii) Annealing rates increase linearly with increasing unlabeled DNA concentration, both without (left) and with (right) 40 nM Twinkle (mean *N* = 2; Supplementary Fig. S4). Linear fits yield bimolecular rate constants. (iii) Bar chart summarizing DNA:DNA bimolecular rate constants with and without Twinkle (±SE from fitting). (**C**) RNA:DNA annealing. (i) Representative kinetics under conditions analogous to DNA annealing (panel B i). (ii) Annealing rates increase linearly with increasing unlabeled DNA concentration, similar to DNA (panel B ii). (iii) Bar chart summarizing RNA:DNA bimolecular rate constants with and without Twinkle (±SE from fitting). (**D**) RNA:RNA annealing. (i) Representative kinetics under conditions analogous to DNA annealing (panel B i). (ii) Annealing rates increase linearly with increasing unlabeled RNA concentration, similar to DNA (panel B ii). (iii) Bar chart summarizing RNA:RNA bimolecular rate constants with and without Twinkle (±SE from fitting).

In addition to the canonical ATPase and unwinding activities, Twinkle has non-canonical DNA annealing and DNA strand-exchange activities [8, 9]. We investigated whether Twinkle could catalyze these reactions on RNA. We developed a real-time fluorescence-based assay to measure the rapid kinetics of nucleic acid annealing activity of Twinkle using a stopped-flow instrument. In this assay, the 28-nt DNA or RNA strand was labeled with fluorescein (FAM) fluorescence at the 3′-end, and the complementary strand contained a stretch of four dG or rG residues. Upon annealing, the FAM label is brought into proximity with the dG/rG residues, resulting in a decrease in fluorescence (Fig. 4A) through an electron transfer mechanism reported previously [41].

In the absence of Twinkle, the FAM-labeled DNA at 10 nM anneals to its complementary DNA at 40 nM with a slow rate (0.001 s^−1^), but in the presence of Twinkle, the DNA:DNA annealing was accelerated by 330-fold (0.33 s^−1^) [Fig. 4B (i) and Supplementary Fig. S4A–H]. A more detailed investigation of the annealing activity showed that the DNA:DNA annealing rate increases linearly with increasing concentration of the complementary unlabeled strand [Fig. 4B (ii)], indicating that DNA annealing is limited by the two strands coming together. The slope provided a bimolecular rate constant of annealing, which was 74-fold greater in the presence of Twinkle than in its absence [Fig. 4B (iii)]. These data confirm our previous findings of DNA:DNA annealing activity of Twinkle while providing quantitative measurements of the activity.

Similar experiments with 28-nt RNA and complementary DNA demonstrated that Twinkle also possesses RNA:DNA annealing activity. Measurement of the kinetics showed that Twinkle accelerates the rate of RNA:DNA annealing by 325-fold compared to the spontaneous rate [Fig. 4C (i) and Supplementary Fig. S4B and H]. The annealing rate increased with increasing concentration of the complementary DNA [Fig. 4C (ii)], providing a bimolecular RNA:DNA annealing rate constant that was ~197-fold greater in the presence of Twinkle compared to the spontaneous rate constant [Fig. 4C (iii)]. The addition of different nucleotide states, ATP, ATPγS, or ADP, did not affect the annealing activity of Twinkle (Supplementary Fig. S4I).

Finally, we evaluated whether Twinkle could anneal complementary RNA strands. Spontaneous RNA annealing was highly inefficient, but the presence of Twinkle greatly increased the RNA:RNA annealing rate by 630-fold [Fig. 4D (i)]. The reaction showed a linear dependence on RNA concentration, yielding a bimolecular rate constant ~2260-fold higher in the presence of Twinkle than the spontaneous rate [Fig. 4D (ii) and (iii)].

Our findings demonstrate that Twinkle is highly effective at annealing not only complementary DNA strands but also RNA:DNA and RNA:RNA. The nucleic acid annealing activity is a unique property of Twinkle, as the homologous T7 gp4 lacks RNA:DNA annealing activity (Supplementary Fig. S4J). Although the exact role of the annealing functions of Twinkle is not understood, the RNA:DNA annealing function may contribute to the formation of R-loops in mitochondria [28]. In the T7 phage, the ssDNA-binding protein gp2.5 has been reported to facilitate DNA annealing and strand-exchange reactions, thereby supporting DNA recombination and repair functions [42–45]. The finding that Twinkle has RNA:RNA annealing activity suggests that it may have yet unexplored RNA chaperone functions.

### Twinkle integrates RNA into dsDNA through toehold-mediated strand exchange

**Figure 5.**
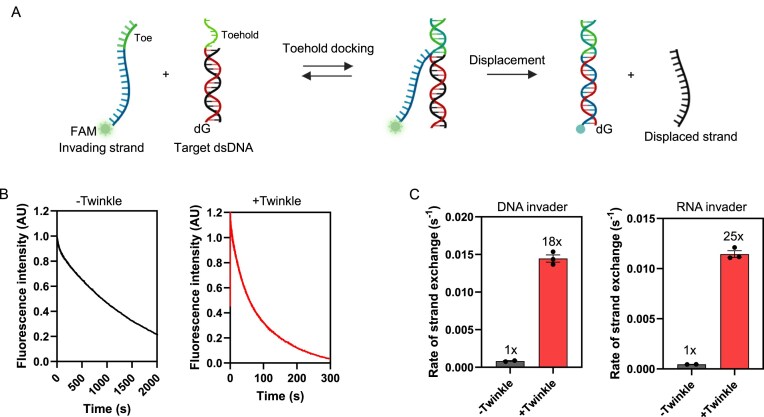
Twinkle facilitates RNA:DNA hybrid formation through toe-mediated strand exchange. (**A**) Schematic of the TMSD reaction. A FAM-labeled DNA or RNA invader anneals its toehold region (green) to the complementary toehold on the target DNA (green), followed by branch migration that displaces the target DNA strand. Completion of the reaction results in quenching of the FAM fluorescence by dG residues in the target, enabling real-time measurement of TMSD kinetics. (**B**) Normalized stopped-flow fluorescence traces showing spontaneous and Twinkle-catalyzed TMSD reactions (see Supplementary Fig. S5 for raw traces). Reactions were performed with 10 nM target dsDNA and 40 nM FAM-labeled invader, in the absence or presence of 40 nM Twinkle hexamer. (**C**) Bar charts summarizing rates of spontaneous and Twinkle-catalyzed TMSD for DNA invader (left) and RNA invader (right). Data represent mean ± SE, *N* ≥ 2.

In addition to its DNA annealing activity, Twinkle catalyzes DNA strand-exchange between complementary ssDNA (invader) and dsDNA (target) [9]. This reaction relies on the presence of a short (>1 nt) toehold in the target dsDNA, which allows the invader to dock, form a joint molecule, and initiate strand-exchange through a branch migration process (Fig. 5A). Twinkle catalyzes this TMSD reaction in the absence of ATP hydrolysis and hence does not require its translocation activity [9]. We investigated whether Twinkle could catalyze TMSD using RNA as the invader strand.

We used a 21-bp target dsDNA with a 7-nt toehold region and a complementary 28-nt RNA, labeled with 3′-FAM, to monitor the TMSD reaction in real time. A parallel reaction with the same sequence DNA invader was conducted for comparison. The 5′-end of the target DNA included four dG residues that, upon completion of the TMSD reaction, come into proximity with the FAM fluorophore, resulting in fluorescence quenching (Fig. 5A). The fluorescence change due to the TMSD reaction was monitored with millisecond time resolution on a stopped-flow instrument. Mixing 10 nM target DNA with 40 nM RNA or DNA invader resulted in a rapid decrease in FAM fluorescence in the presence of Twinkle, which indicated successful annealing of the invader (DNA or RNA) to the target DNA through TMSD (Fig. 5B and Supplementary Fig. S5).

The TMSD reaction also occurred spontaneously, but at a very slow rate (8.2 × 10^−4^ s^−1^ with the DNA invader and 4.5 × 10^−4^ s^−1^ with the RNA invader). However, the addition of Twinkle increased the TMSD rate of the DNA invader by ~18-fold (0.015 s^−1^) and the RNA invader by ~25-fold (0.011 s^−1^) (Fig. 5C). These findings demonstrate that Twinkle can catalyze TMSD with RNA and hence promote the invasion of an RNA strand into a complementary region in the dsDNA to generate an RNA:DNA hybrid or an R-loop.

### Twinkle’s RNA strand-exchange activity can rescue a stalled replication fork

The RNA:DNA hybrids can have both harmful and beneficial effects on genome integrity [46–48]. If unresolved, R-loops can lead to DNA breaks and unregulated DNA replication, compromising genomic stability. On the other hand, the R-loop may be used in restarting a stalled replication fork. In this process, the invading RNA serves as a primer to facilitate replication restart downstream of a stalled replication fork [49]. Mitochondria lack recombination and repair proteins that the nucleus uses for genome integrity. Interestingly, Twinkle has been implicated in the rescue of stalled replication forks in mitochondria of cultured cells, but the mechanism is not clearly understood [17].

**Figure 6.**
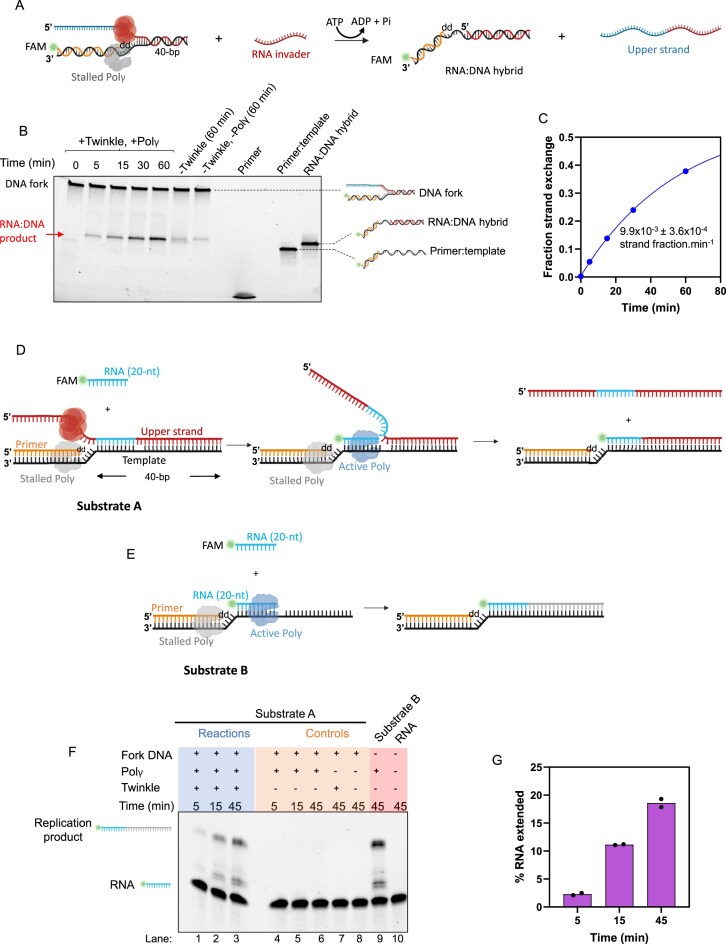
Twinkle-mediated RNA invasion rescues stalled replication fork. (**A**) Schematic of RNA invader-mediated unwinding/strand-exchange, forming an RNA:DNA hybrid at a stalled replication fork. (**B**) Representative 4%–20% native PAGE showing time course of RNA:DNA product formation (*N* = 2). Reactions were initiated by adding RNA invader and MgATP to stalled fork substrates pre-assembled with Polγ and Twinkle. Controls were run for 60 min. (**C**) Kinetics of RNA:DNA product from panel (B) fitted to eq. (4); initial strand-exchange rates were determined using eq. (6) (mean ± SEM, *N* = 3). (**D**) Experimental design for Twinkle-mediated rescue of a stalled replication fork (Substrate A). Twinkle-mediated unwinding/strand-exchange activity introduces a FAM-RNA primer at the stalled fork, which Polγ then uses to reinitiate DNA synthesis. (**E**) Control reaction with Substrate B pre-annealed to the FAM-RNA primer provides a marker for the expected length of product by Polγ. (**F**) Representative 15% denaturing urea PAGE showing RNA extension (*N* = 2). Reactions initiated with FAM-RNA, dNTPs, and MgATP (lanes 1–3). Controls include reactions without Twinkle (lanes 4–6), without Polγ (lane 7), without both Polγ and Twinkle (lane 8), and Substrate B with Polγ (lane 9). Lane 10 shows the FAM-primer. (**G**) Fraction of extended replication products quantified from lanes 1–3 in panel (G) (mean *N* = 2).

We designed an *in vitro* assay to investigate whether Twinkle can anneal an RNA primer downstream of a stalled replication fork. A stalled replication fork was created by assembling Twinkle and Polγ on a replication fork with a non-extendable ddCMP at the 3′-end of the DNA primer (Fig. 6A). Subsequently, an RNA primer complementary to the downstream duplex DNA region of the fork was added. Because there is no toehold region in the fork substrate, Twinkle must unwind the DNA to create a toehold, allowing the TMSD reaction to occur with the RNA primer. Hence, this reaction required the presence of MgATP. In the absence of Twinkle, no significant strand-exchange product was observed. However, in the presence of Twinkle, ~40% of the fork DNA was annealed to the RNA primer. The strand-exchange reaction occurred with an initial rate of 0.01 strand fraction.min^−1^ (Fig. 6B and C). To clearly demonstrate the RNA exchange, we also loaded the control substrates next to the reaction products and used two different percentages of polyacrylamide, which demonstrates that Twinkle indeed catalyzes the RNA invasion reaction (Supplementary Fig. S6).

We next designed an experiment to investigate whether Polγ could use the newly exchanged RNA primer to reinitiate DNA synthesis. To test this, the invader RNA was labeled with 5′-end FAM to monitor its extension and was designed to be 20-nt shorter than the dsDNA region of the fork substrate. Strand-exchange would create an RNA:DNA hybrid at the stalled replication fork with a 20-bp downstream duplex template region for RNA primer extension by Polγ (Substrate A) (Fig. 6D). A substrate with a pre-annealed FAM-RNA primer was used as a positive control for primer extension by Polγ (Substrate B, Fig. 6E). Controls without Twinkle or without Polγ did not show any RNA extension (Fig. 6F), which indicates that Twinkle is required for RNA invasion and that RNA does not spontaneously undergo strand-exchange. It also confirmed that our reaction mixture did not contain single-stranded template DNA. RNA extension was observed when both Twinkle and Polγ were present (Fig. 6F). After a 45-min reaction, ~20% of the RNA primer was extended to the full-length product (Fig. 6G). A similar length of the extended product was observed in the positive control (Substrate B) that contained a pre-annealed FAM-RNA primer.

These experiments demonstrate that Twinkle can use its RNA strand-exchange activity to anneal an RNA to a complementary region in the dsDNA, and Polγ can extend the RNA for DNA synthesis. In addition to rescuing stalled forks, the annealing/strand-exchange activity of Twinkle could also play a role in the formation of R-loops or RNA:DNA hybrids for the initiation of mtDNA replication [50].

### Effect of mtSSB on the non-canonical activities of Twinkle

Because mtSSB is an integral component of the mt replisome, we asked whether it modulates the non-canonical annealing and TMSD activities of Twinkle. We first examined DNA:DNA and RNA:DNA annealing in the presence of increasing concentrations of mtSSB. As mtSSB concentration increased, Twinkle-mediated annealing of both DNA:DNA and RNA:DNA duplexes was progressively inhibited (Supplementary Fig. S7A–C). This inhibition is most likely due to competition for nucleic acid binding, as both mtSSB and Twinkle bind ssDNA and RNA with comparable affinities. Because nucleic acid binding by Twinkle is required for annealing, occupancy of the nucleic acid substrates by mtSSB reduces the efficiency of Twinkle-mediated annealing.

Unexpectedly, we observed a biphasic effect of mtSSB on the Twinkle-catalyzed TMSD reaction using either DNA or RNA invaders. Low concentrations of mtSSB stimulated TMSD, whereas higher concentrations inhibited the reaction (Supplementary Fig. S7D–F). We speculate that the initial activation reflects mtSSB’s ability to resolve secondary structures within the invader strand (Supplementary Fig. S7G), thereby promoting its annealing to the 7-nt toehold region of the target DNA. At higher levels, inhibition likely arises from competition between mtSSB and the invader strand for substrate binding. Consistent with this, mtSSB also inhibited the replication-restart activity of Twinkle and Polγ using an RNA primer (Supplementary Fig. S7H–J).

Together, these results suggest that mtSSB regulates Twinkle’s non-canonical activities by suppressing RNA:DNA hybrid formation and RNA-primed replication restart. The competitive inhibition by mtSSB observed here may be specific to short oligonucleotide substrates. Therefore, future studies using mt DNA-length substrates will be necessary to define how mtSSB regulates these non-canonical processes in a more physiologically relevant context.

### W315L mutation in Twinkle impairs replication but not RNA:DNA hybrid formation

It is remarkable that a replicative helicase like Twinkle has evolved opposing activities, enabling it to both unwind DNA and anneal/exchange nucleic acid strands. However, an intricate balance of the helicase and strand annealing/exchange activities of Twinkle would be required to maintain a healthy mt genome. Twinkle variants have been linked to diseases characterized by mtDNA deletions and reduced copy number [3, 4, 20, 51, 52]. To explore whether these Twinkle mutations disrupt the balance between Twinkle’s unwinding and annealing functions, we focused on a well-characterized disease-associated variant, W315L. The W315L mutation resides in the N-terminal domain of Twinkle, near the flexible linker connecting it to the C-terminal domain. This mutation alters Twinkle’s oligomerization state, shifting it from predominantly hexameric assemblies, as seen in the wild type, to higher-order oligomers such as heptamers and octamers [35, 53]. To understand how the W315L mutation affects Twinkle’s function, we examined its impact on DNA replication, nucleic acid binding, nucleic acid annealing, and strand-exchange activities.

**Figure 7.**
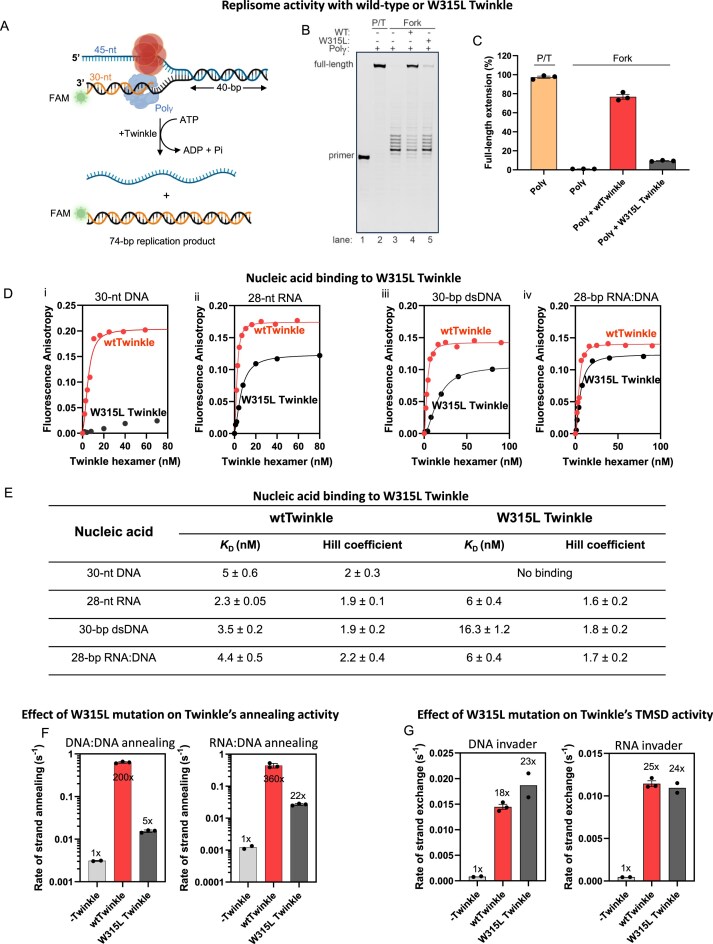
W315L Twinkle is defective in DNA replication but competent in strand-exchange. (**A**) Schematic of strand displacement DNA synthesis by Twinkle and Polγ on a 40-bp forked DNA with a 45-nt 5′-tail and 34-nt 3′-tail annealed to a 30-nt FAM-primer, creating a 4-nt gap between primer end and downstream duplex. (**B**) Representative denaturing PAGE (*N* = 3) showing primer extension by Polγ on primer:template (P/T, lane 2) or fork DNA in the absence of Twinkle (lane 3), with wild-type Twinkle (lane 4), or W315L Twinkle (lane 5). (**C**) Quantification of full-length primer extension product from panel (B) (mean ± SEM, *N* = 3). (**D**) Fluorescence anisotropy titrations measuring binding of wild-type or W315L Twinkle to FAM-labeled (i) DNA, (ii) RNA, (iii) dsDNA, and (iv) RNA:DNA hybrid (mean *N* = 2). Solid lines are fits to eq. (1). (**E**) Table summarizing *K*_D_ values of W315L Twinkle and wtTwinkle (from Fig. 2G) for various nucleic acid substrates. (**F**) Bar charts comparing DNA and RNA annealing rates by wild-type and W315L Twinkle to spontaneous reactions. Reactions contained 10 nM FAM-labeled ss nucleic acid, 80 nM unlabeled complementary ssDNA, and 40 nM wild-type or W315L Twinkle. (**G**) Bar charts showing TMSD rates with 10 nM target dsDNA, 40 nM wild-type or W315L Twinkle, and 40 nM DNA (left) or RNA (right) invader.

To evaluate whether the W315L mutation impacts Twinkle’s replisome function, we conducted DNA synthesis assays using primed 40-bp DNA fork substrate (Fig. 7A). The primer extension products from these reactions were analyzed on a denaturing gel (Fig. 7B). Polγ alone showed minimal strand displacement synthesis on the fork substrate (Fig. 7B). In contrast, wild-type Twinkle and Polγ extended ~80% of the primer substrate to the full-length product (Fig. 7B and C). However, with W315L Twinkle, Polγ extended only ~10% of the primer to the full-length product. Thus, the W315L mutation significantly impairs Twinkle’s ability to support strand displacement DNA synthesis by Polγ.

We next examined the nucleic acid binding, annealing, and strand-exchange functions of W315L Twinkle with both RNA and DNA substrates (Fig. 7D and Supplementary Fig. S8). Fluorescence anisotropy-based measurements revealed that W315L has a very weak affinity for ssDNA, with only a small increase in fluorescence anisotropy observed with increasing protein concentration. On the other hand, W315L showed reasonable binding affinity to RNA, as well as dsDNA and RNA:DNA hybrid [Fig. 7D (ii)–(iv) and E].

W315L catalyzed DNA:DNA and RNA:DNA annealing reaction, but with a lower efficiency compared to wild-type Twinkle (Fig. 7F). W315L accelerated the spontaneous strand annealing reactions of DNA:DNA and RNA:DNA at 80 nM concentration by 5- and 22-fold, respectively, compared to the 200- and 360-fold increases observed with the wild-type Twinkle under similar nucleic acid concentrations (Fig. 7F).

Interestingly, W315L retained robust RNA and DNA strand-exchange activity through the TMSD mechanism, comparable to wild-type Twinkle (Fig. 7G). We speculate that the reduced DNA and RNA annealing activity may be due to the weaker ssDNA affinity of W315L compared to wild type, while the TMSD catalysis remains unaffected because W315L binds relatively well to dsDNA. These findings indicate that the W315L mutation impacts the canonical DNA replication-related activities but not the non-canonical strand-exchange activities with DNA or RNA.

## Discussion

Human mt helicase Twinkle has evolved distinct functional modalities as compared to bacteriophage T7 gp4, its ancestral homolog. The human Twinkle has lost its primase function and acquired functions, including DNA annealing and strand-exchange activities, which are absent in gp4. In this study, we extend Twinkle’s repertoire by demonstrating that it binds RNA and RNA:DNA hybrids with high affinity. Furthermore, Twinkle can generate RNA:DNA hybrids either by annealing ssRNA and DNA or by replacing a DNA strand in a duplex with a complementary RNA strand, through its ATPase-driven unwinding activity or by the TMSD mechanism. Twinkle can also anneal complementary RNA strands, which suggests its role in RNA-related activities.

The physiological relevance of Twinkle’s RNA-related activities remains unclear. However, recent findings have localized Twinkle in mtRNA granules, where it contributes to their stability [23]. These granules are RNA-rich foci that support essential post-transcriptional processes, including RNA processing, modification, ribosome assembly, and degradation [54]. Based on our results, we propose that Twinkle may serve dual functions in mitochondria: driving DNA replication in concert with Polγ on mtDNA in nucleoids, while also supporting RNA maturation processes within RNA granules. To our knowledge, Twinkle has not yet been directly linked to any specific RNA maturation pathway. Given that ATP hydrolysis is not required or has any effect on the non-canonical activities, we currently favor the idea that Twinkle may act not as a helicase in the RNA-related functions, but rather as an RNA chaperone. Further studies will be needed to confirm such RNA-related functions of Twinkle.

Our data further reveal that Twinkle can catalyze the invasion of RNA into duplex DNA, suggesting a role in mt R-loop biology. R-loops are abundant in mitochondria, as first reported in mouse studies [55] and subsequently confirmed in human cells, where both transient and RNase H-resistant R-loops have been detected [26, 27, 29, 56]. R-loops are also crucial for the initiation of mtDNA replication [50]. Notably, Twinkle depletion reduces mt RNA:DNA hybrids [23], supporting its role in the formation or stabilization of R-loops. We show that Twinkle’s strand-exchange activity can anneal an RNA primer downstream of a stalled replication fork, enabling Polγ to resume DNA synthesis. This activity provides a plausible mechanism by which Twinkle could facilitate replication reinitiation following fork stalling. Additionally, RNA invasion activity could play a role in the initiation of mtDNA replication by providing primers for DNA synthesis. Interestingly, our studies indicate that mtSSB can inhibit the formation of RNA:DNA hybrids and the restart of RNA primer replication. As RNA:DNA hybrids can affect many mt processes, the interplay between mtSSB and Twinkle in RNA:DNA hybrid formation, revealed by our initial studies, requires further investigation in future studies.

Twinkle’s annealing activity may explain RNA:DNA hybrids observed in replication intermediates. Mitochondrial DNA replication proceeds via an asynchronous mechanism that generates long stretches of single-stranded DNA, which are coated by mtSSB [1, 57] or hybridized with processed transcripts [25, 58, 59]. We previously reported Twinkle’s DNA annealing activity [8] that raised the possibility that it participates in creating RNA:DNA hybrids at these regions. The present study supports this hypothesis by demonstrating that Twinkle can indeed catalyze the annealing of RNA and DNA.

Ultimately, our findings have implications for human health and disease. Mutations in Twinkle cause mtDNA deletions and depletion syndromes, including progressive external ophthalmoplegia, neurodegeneration, and Perrault syndrome [4]. We find that a pathogenic variant W315L, while defective in DNA replication, retains annealing and strand-exchange activities. In the nucleus, R-loops function as double-edged regulators, both harmful and beneficial, influencing transcription, replication, and genome stability [46–48, 60]. By analogy, Twinkle-mediated R-loops may similarly have dual roles in the mt genome. However, in the context of impaired replication in Twinkle variants, accumulation of RNA:DNA hybrids could compromise genome integrity. Since mtDNA deletions often arise from repeat sequence misannealing events during error-prone repair or copy-choice recombination [21, 22], we propose that the imbalance between replication and annealing/strand-exchange activity in W315L may contribute to mtDNA deletions. These findings open avenues for investigating the pathological role of Twinkle’s non-canonical functions and raise the possibility of therapeutic strategies that target these activities.

### Limitations of the study

Our findings expand the functional repertoire of Twinkle and suggest that, beyond its canonical role in DNA replication, its DNA and RNA annealing and strand-exchange activities may contribute to mtRNA metabolism, R-loop regulation, replication initiation and restart, and potentially pathological mtDNA rearrangements. We also find that mtSSB suppresses these activities. However, these non-canonical functions and the effect of mtSSB were identified through *in vitro* biochemical assays on short RNA and DNA oligonucleotide substrates, which may not fully capture the structural and physiological complexity of mt nucleic acids. Future work should evaluate these activities on longer templates or closed circular DNA substrates that more closely resemble the mt genome. Additionally, in-cell studies are needed to characterize Twinkle’s RNA-binding activity under physiological conditions and to systematically define its contributions to RNA processing and metabolism *in vivo*.

## Supplementary Material

gkag008_Supplemental_File

## Data Availability

The data underlying this article are available in the article and in its online supplementary material.
