# Supplementary material for: Human mitochondrial helicase Twinkle has RNA binding, annealing, and strand-exchange activities
Source: Nucleic Acids Res. 2026 Jan 21;54(2):gkag008. doi: 10.1093/nar/gkag008 (PMC12820530; doi:10.1093/nar/gkag008)
Supplement: gkag008_Supplemental_File [file gkag008_supplemental_file.pdf]

Supplementary Fig. S1

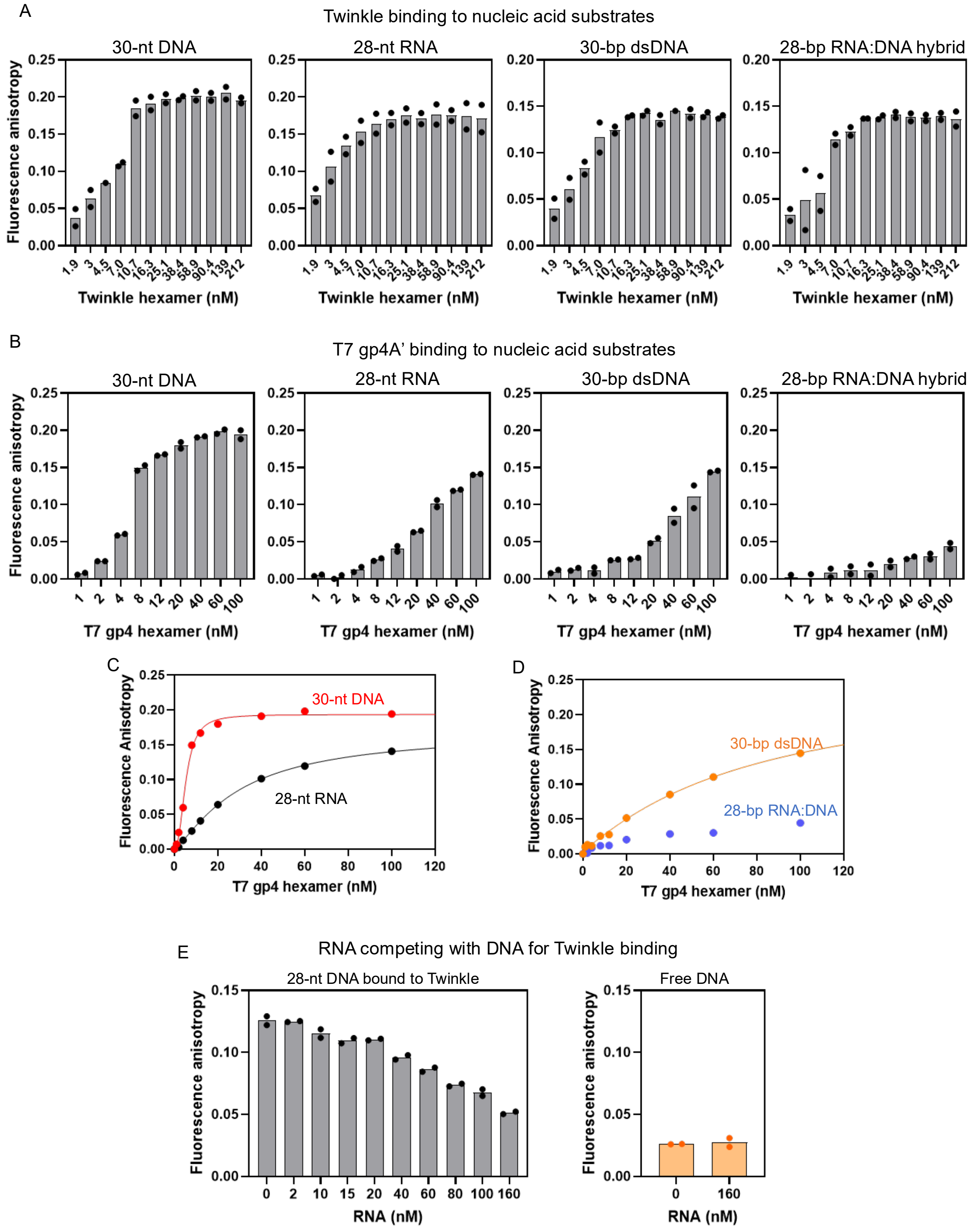

Supplementary Fig. S1. Twinkle's binding to various nucleic acid substrates.

(**A**) Fluorescence anisotropy measurements of various FAM-labeled nucleic acid substrates in presence of different Twinkle concentrations (mean, $N = 2$). (**B**) Fluorescence anisotropy measurements of various FAM-labeled nucleic acid substrates in presence of different concentrations of bacteriophage T7 gp4A' helicase (mean, $N = 2$). (**C**) Fluorescence anisotropy was measured by mixing FAM-labeled single-stranded or (**D**) double-stranded nucleic acid substrates with different T7 gp4 concentrations. The data were fit to *Equation 1* to estimate the dissociation constants. The data points are means of two independent measurements. (**E**) 40 nM FAM-labeled 28-nt ssDNA was added to increasing concentrations of unlabeled 28-nt RNA in presence (left panel, gray bars) and in absence (right panel, orange bars) of 40 nM Twinkle hexamer. Bars represent means from two independent reactions.

Supplementary Fig. S2

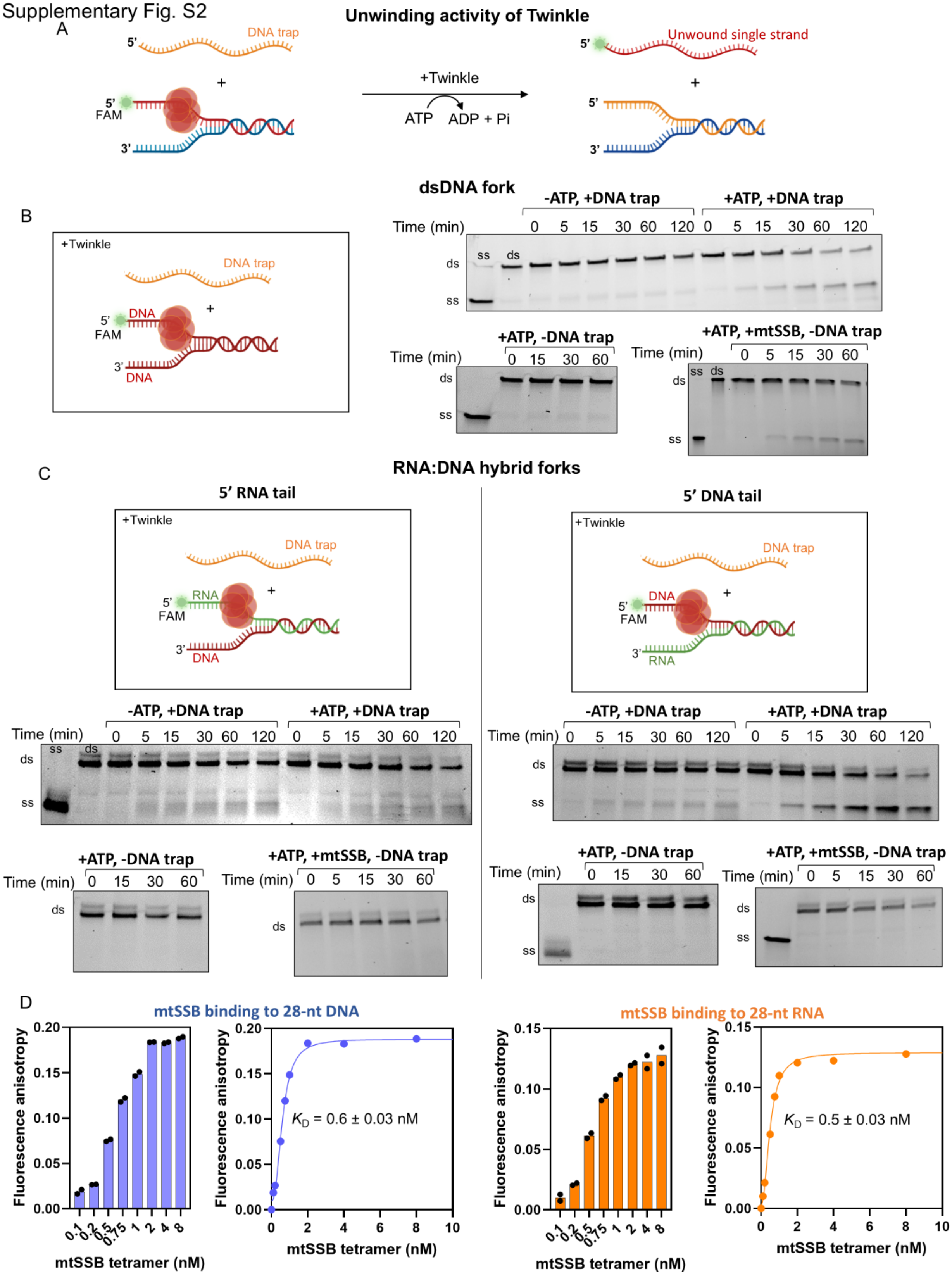

Supplementary Fig. S2. Twinkle's unwinding activity tested on dsDNA or RNA:DNA hybrid forks.

(**A**) Schematics of experimental design to test Twinkle's unwinding/strand exchange activity on fork substrate. (**B**) Unwinding reactions were performed on a dsDNA fork and reaction products were resolved on 4-20% native PAGE. Reactions were performed with DNA trap in presence and absence of MgATP (top) and presence of MgATP but without DNA trap (bottom, left) or with MgATP and 10 nM mtSSB tetramer (bottom, right). The gels images are representatives of the duplicates. (**C**) Twinkle-catalyzed unwinding reactions performed on RNA:DNA hybrid forks with 5' RNA-tail (left) and 5' DNA-tail (right) and reaction products analyzed on 4-20% native PAGE. Reactions were performed under various conditions as described in B. Gel images are representative of the two independent repeats. (**D**) MtSSB binds to RNA. Fluorescence anisotropies of 2.5 nM FAM-labeled 28-mer DNA or 28-mer RNA measured as a function of mtSSB concentration. Bar charts show means from two repeats (mean, $N = 2$). The means of the two replicates were fit to *Equation 1*, providing dissociation constants. Errors depicted are standard errors from data fitting.

Supplementary Fig. S3

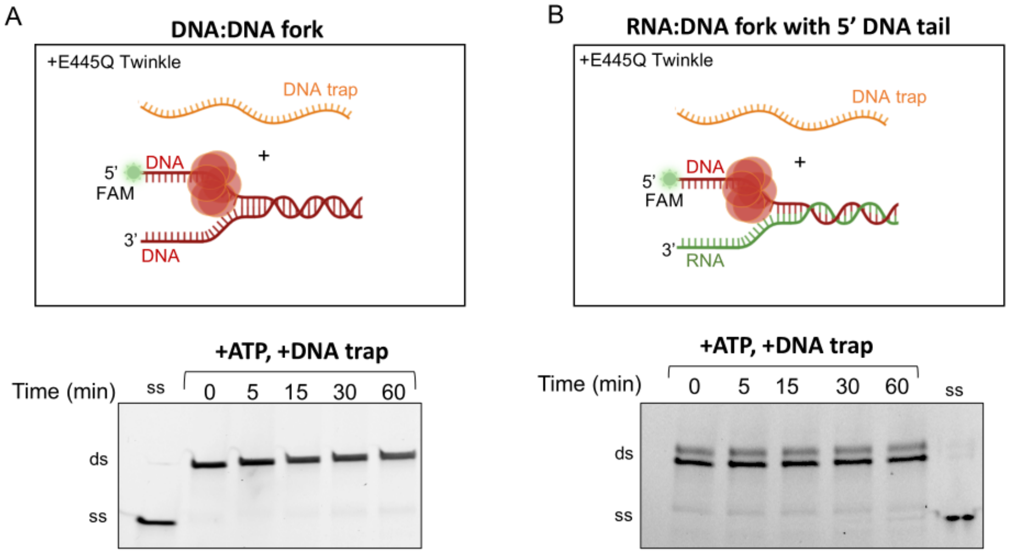

Supplementary Figure S3. Effect of E44Q mutation on Twinkle's unwinding/strand exchange activity.

(**A-B**) Unwinding of DNA:DNA (A) and DNA:RNA (B) forks (with 5'-DNA tail) by E445Q Twinkle. Reactions contained 10 nM fork substrate, 55.5 nM E445Q Twinkle hexamer, and 100 nM complementary DNA trap at 25 °C. The reactions were resolved by native PAGE.

Supplementary Fig. S4

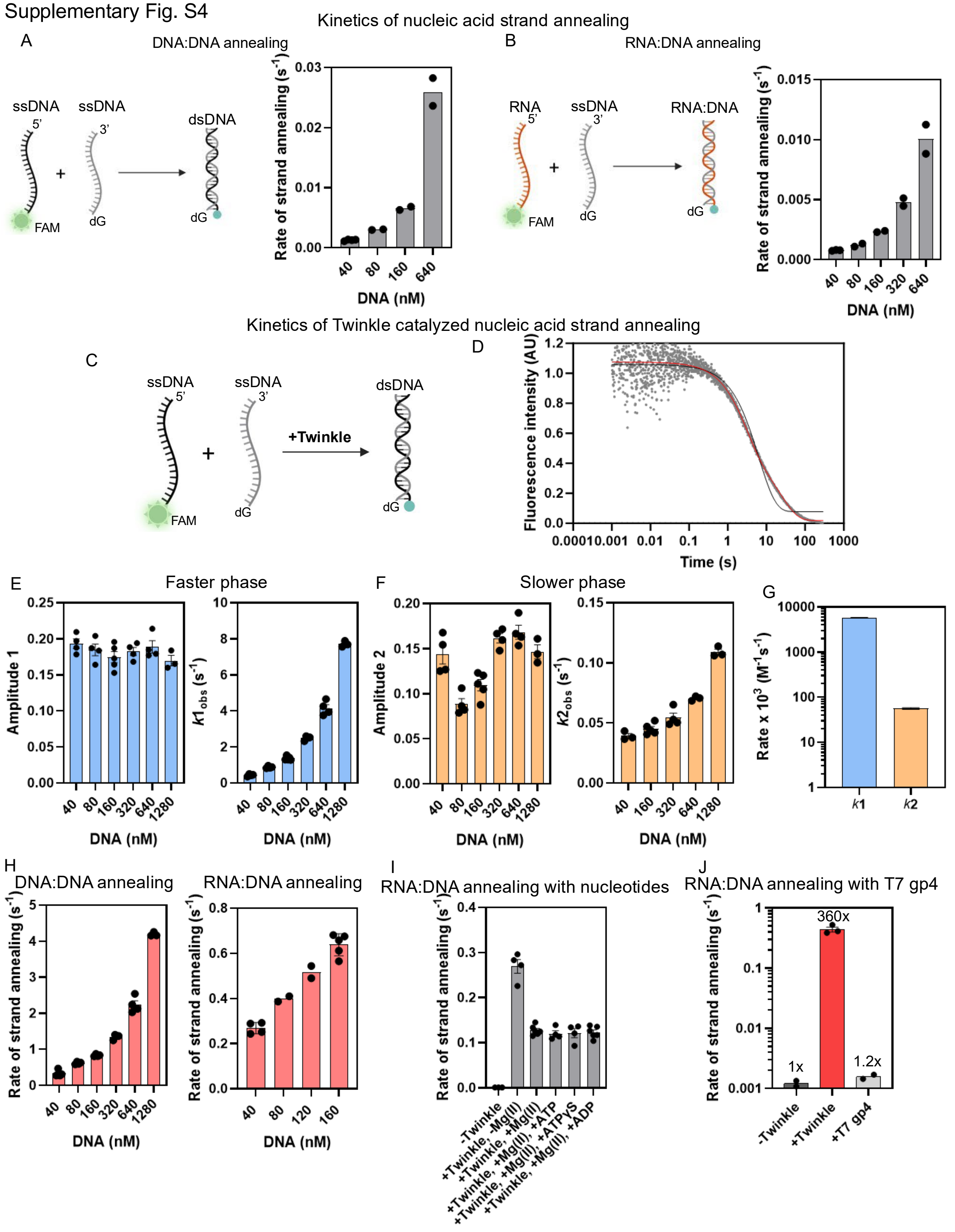

Supplementary Fig. S4. Kinetics of Twinkle catalyzed strand annealing.

(**A-B**) 10 nM FAM-labeled ssDNA or RNA was mixed with increasing concentrations of complementary ssDNA containing four dG residues which quench FAM fluorescence as the two complementary strands anneal. Fluorescence time courses fit to single-exponential *Equation 4*. Observed rates of DNA:DNA (A) and RNA:DNA (B) annealing measured with said concentrations of unlabeled ssDNA. Bars represent means from at least two independent observations (mean, $N \geq 2$). (**C-D**) Schematics of experimental design to measure kinetics of Twinkle-catalyzed strand annealing (C). Representative stopped-flow time trace showing fluorescence decrease due to DNA:DNA annealing (D). Data points fit poorly to a single-exponential *Equation 4* (black line) and were fit to a double-exponential *Equation 5* (red line) to improve fitting. (**E-F**) Observed amplitudes and rates from faster (E) and slower (F) kinetic phases of Twinkle-catalyzed DNA annealing as obtained from fits of stopped-flow fluorescence traces to double-exponential *Equation 5*. Bar charts show means from at least three independent annealing reactions with error bars showing standard errors of means (mean ± SEM, $N \geq 3$). (**G**) Bimolecular DNA:DNA annealing rates of fast ($k1$) and slow ($k2$) kinetic phases obtained from slopes of linear fits of kinetic data in E and F, respectively. (right panels). Error bars represent standard errors from fitting. (**H**) Rates of Twinkle-catalyzed DNA:DNA (left) and RNA:DNA (right) strand annealing reactions performed with 10 nM FAM-labeled ssDNA or RNA and increasing concentrations of the unlabeled complementary ssDNA. Bars show means from at least two independent annealing reactions (mean ± SEM, $N \geq 2$). (**I**) Effect of magnesium ions and nucleotides on kinetics of Twinkle's RNA:DNA annealing activity (mean ± SEM, N ≥ 3). Reactions contained 10 nM FAM-labeled RNA, 40 nM complementary ssDNA containing dG residues and 40 nM Twinkle. (**J**) Bar chart showing observed rates of RNA:DNA annealing in presence of Twinkle or T7 gp4. The reactions were performed with 10 nM FAM-labeled ssDNA or RNA, 80 nM complementary ssDNA containing dG residues and 40 nM Twinkle or T7 gp4 hexamers. Reactions with T7 gp4 contained 1 mM dTTP to induce nucleic acid binding. Bars show means from at least two independent reactions (mean ± SEM, N ≥ 2).

Supplementary Fig. S5

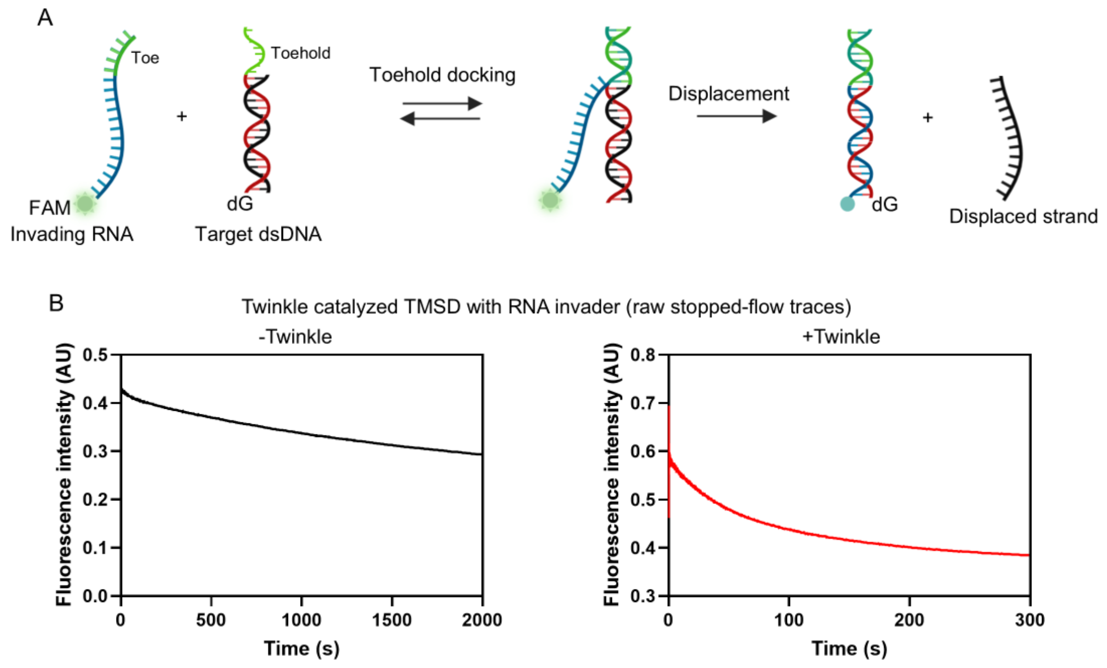

Supplementary Figure S5. Stopped flow assay to monitor Twinkle's facilitation of RNA:DNA hybrid formation through toe-mediated strand exchange.

(A) Schematic of the TMSD reaction. (**B**) Raw stopped-flow fluorescence traces showing spontaneous and Twinkle-catalyzed TMSD reactions. These traces were normalized (normalized traces are shown in Figure 5). Reactions were performed with 10 nM target dsDNA and 40 nM FAM-labeled invader, in the absence or presence of 40 nM Twinkle hexamer.

Supplementary Fig. S6

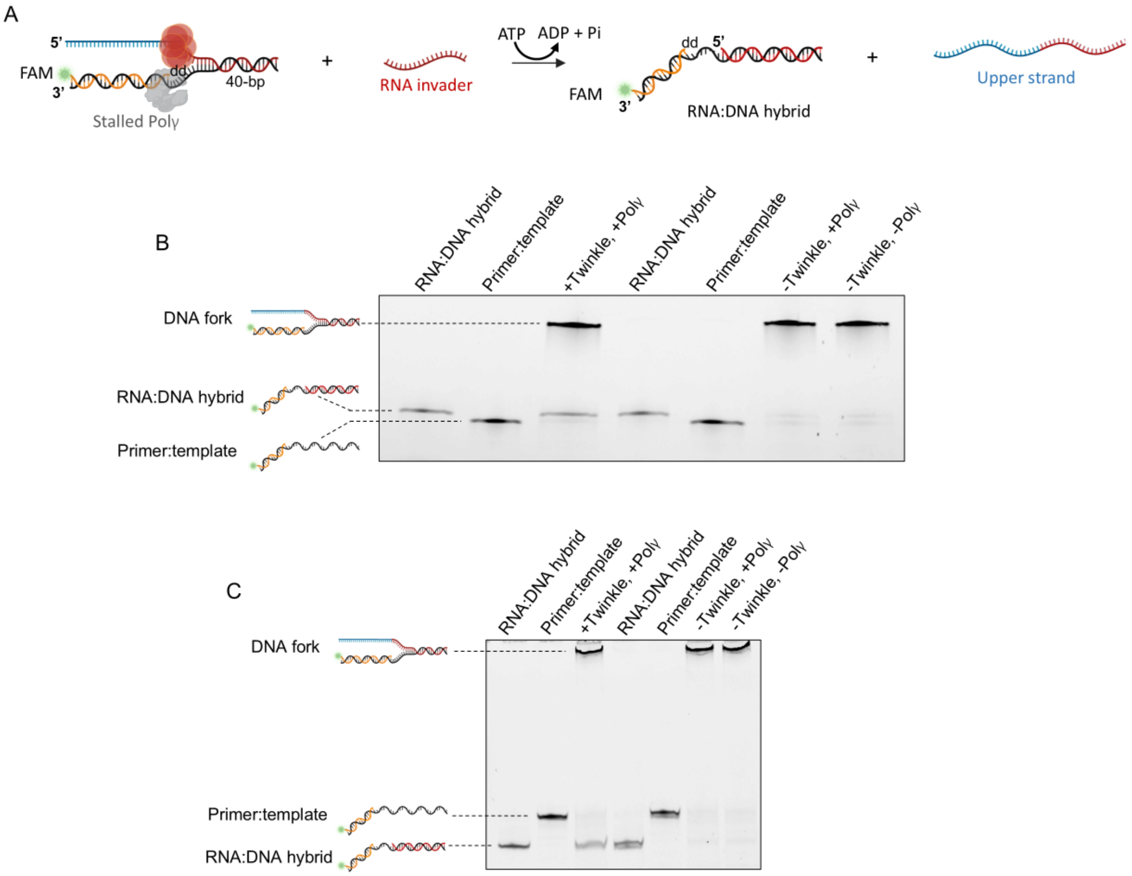

Supplementary Figure S6. Twinkle facilitates RNA invasion on stalled replication fork.

(**A**) Schematic of RNA invader-mediated unwinding/strand exchange, forming an RNA:DNA hybrid at a stalled replication fork. (**B-C**) Representative 4–20% (B) and 16% (C) native PAGE showing 30 min reactions with markers (primer-template and RNA:DNA hybrid) and controls.

Supplementary Fig. S7

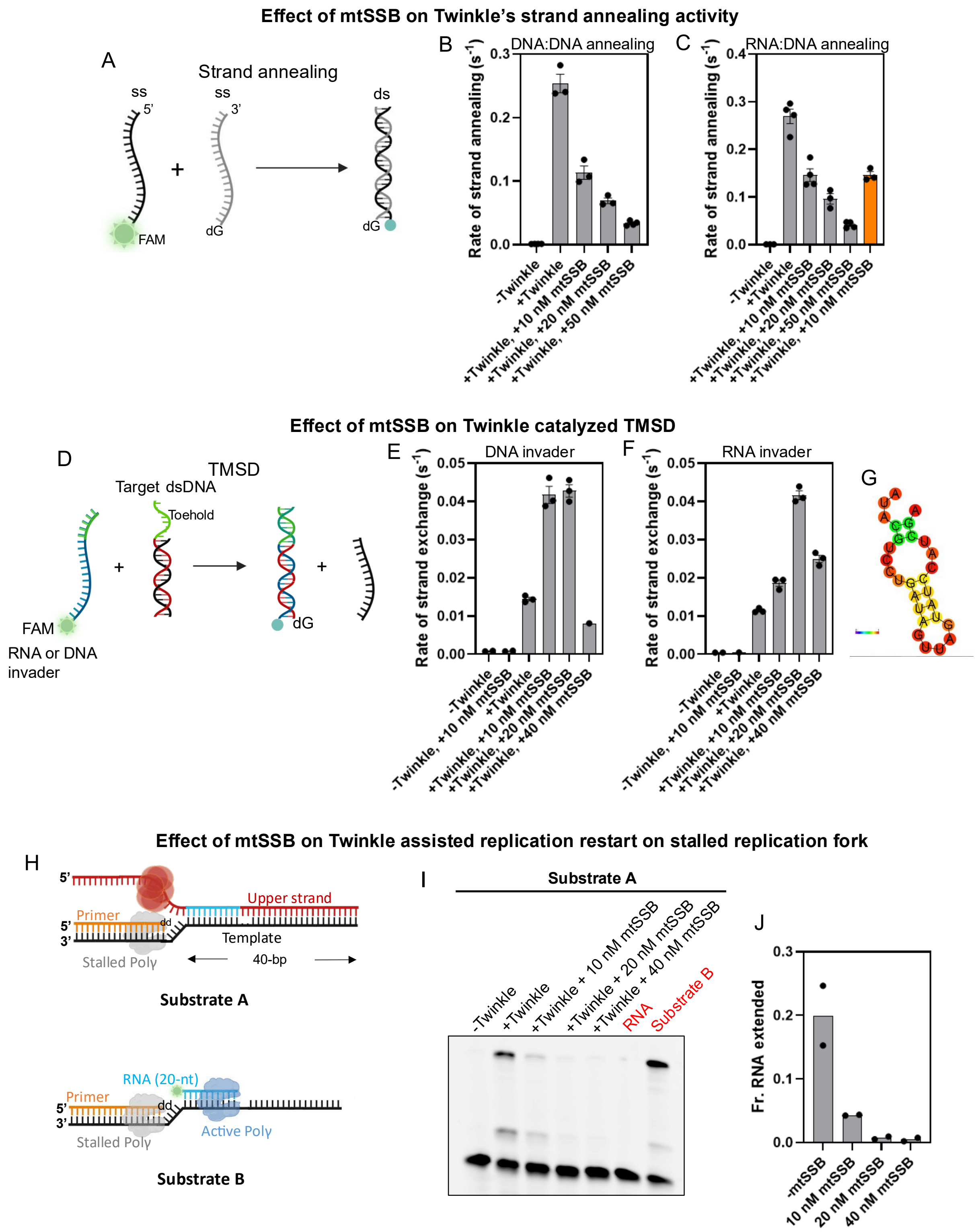

Supplementary Figure S7. Effect of mtSSB on Twinkle's RNA-related activities.

(**A-C**) Schematic of strand annealing reaction (A). Bar chart showing effect of mtSSB on Twinkle catalyzed DNA:DNA (B) and RNA:DNA (C) annealing reactions. The grey bars in C show the reactions performed with FAM-labeled RNA and unlabeled ssDNA while the orange bar shows kinetics of reactions performed with FAM-labeled DNA and an unlabeled complementary RNA. All the reactions contained 10 nM FAM-labeled strand, 40 nM unlabeled strand, 40 nM Twinkle hexamer and different concentrations of mtSSB tetramer. (**D-G**) Schematic of the TMSD reaction (D). Effect of mtSSB on Twinkle catalyzed TMSD reactions performed with 10 nM target dsDNA, 40 nM Twinkle hexamer and 40 nM ssDNA (E) or RNA (F) invader. Predicted secondary structure of the RNA invader (**G**) (predicted on http://rna.tbi.univie.ac.at/cgi-bin/RNAWebSuite/RNAfold.cgi). (**H-J**) Effect of mtSSB on Twinkle catalyzed replication restart. Design of substrates A and B (H). Representative 15% denaturing urea PAGE showing RNA extension (I). Reactions were initiated with FAM-RNA, dNTPs, and MgATP. Different concentrations of mtSSB were added with FAM-RNA. Control includes a reaction without Twinkle and Substrate B with Poly. Fraction of extended replication products quantified from H (mean, N = 2) (J).

Supplementary Fig. S8

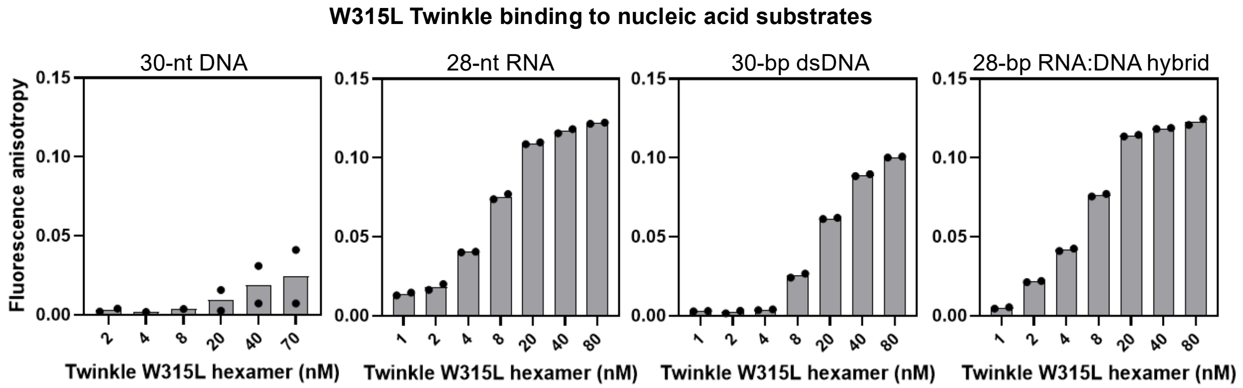

Supplementary Fig. S8. Fluorescence anisotropy measurements of different FAM-labeled nucleic acid substrates with increasing concentrations of Twinkle W315L hexamer (mean, *N* = 2).

Supplementary Table S1

**Oligo ID**	**Sequence**
**Nucleic acid binding reactions**	
30-nt DNA	5’ TGTGTTAGTTGGGGGGTGACTGTTAAAAGT -FAM 3’
DNA complementary to 30-nt DNA	5’ ACTTTTAACAGTCACCCCCCAACTAACACA 3’
28-nt RNA	5’ rArUrArCrGrUrCrCrUrGrArUrArGrUrUrArGrUrArUrCrCrArUrCrGrA-FAM 3’
DNA complementary to 28-nt RNA	5’ GGGGTCGATGGATACTAACTATCAGGACGTAT 3’
27-nt RNA	5’ rArUrArCrGrUrCrCrUrGrArUrArGrUrUrArGrUrArUrCrCrArUrCrG3’
28-nt DNA	5’ ATACGTCCTGATAGTTAGTATCCATCGA -FAM 3’
**Strand-exchange reactions**	
**DNA:DNA fork**	
5’-tail strand	5’ FAM-TTTTTTTTTTTTTTTTTTTTTTTTTTTTTTGCTGGCTATACGTTCACAGTTGACCTACATAGAGTCCTCC 3’
3’-tail strand	5’ GGAGGACTCTATGTAGGTCAACTGTGAACGTATAGCCAGCTTTTTTTTTTTTTTTTTTTTTTTTTTTTTT 3’
Trap DNA	5’ TTTTTTTTTTTTTTTTTTTTTTTTTTTTTTGCTGGCTATACGTTCACAGTTGACCTACATAGAGTCCTCC 3’
**RNA:DNA fork with RNA 5’-tail**	
5’-tail strand	5’ dT FAM- rGrArArUrUrArCrUrUrArArCrArUrCrGrArGrUrGrArUrUrCrCrGrCrArGrArGrCrUrGrGrCrUrArUrArCrGrU<br>rUrCrArCrArGrUrUrGrArCrCrUrArCrArUrArGrArGrUrCrCrUrCrC 3’
3’-tail strand	5’ GGAGGACTCTATGTAGGTCAACTGTGAACGTATAGCCAGCTTTTTTTTTTTTTTTTTTTTTTTTTTTTTT 3’
Trap DNA	5’ GAATTACTTAACATCGAGTGATTCCGCAGAGCTGGCTATACGTTCACAGTTGACCTACATAGAGTCCTCC 3’
**DNA:RNA fork with DNA 5’-tail**	
5’-tail strand	5’ FAM-TTTTTTTTTTTTTTTTTTTTTTTTTTTTTTGCTGGCTATACGTTCACAGTTGACCTACATAGAGTCCTCC 3’
3’-tail strand (70-nt RNA)	5’ rGrGrArGrGrArCrUrCrUrArUrGrUrArGrGrUrCrArArCrUrGrUrGrArArCrGrUrArUrArGrCrCrArGrCrUrCrUrGrCrGrG<br>rArArUrCrArCrUrCrGrArUrGrUrUrArArGrUrArArUrUrC 3’
Trap DNA	5’ TTTTTTTTTTTTTTTTTTTTTTTTTTTTTTGCTGGCTATACGTTCACAGTTGACCTACATAGAGTCCTCC 3’
**Nucleic acid annealing**	
28-nt RNA	5’ rArUrArCrGrUrCrCrUrGrArUrArGrUrUrArGrUrArUrCrCrArUrCrGrA-FAM 3’
28-nt DNA	5’ ATACGTCCTGATAGTTAGTATCCATCGA -FAM 3’
ssDNA complementary to 28-nt RNA and DNA	5’ GGGGTCGATGGATACTAACTATCAGGACGTAT 3’
ssRNA complementary to 28-nt RNA and DNA	5’ rGrGrGrGrUrCrGrArUrGrGrArUrArCrUrArArCrUrArUrCrArGrGrArCrGrUrArU 3’
**TMSD assays**	
Target strand	5’ GGGGTCGATGGATACTAACTATCAGGACGTAT 3’
Displaced strand	5’ CTGATAGTTAGTATCCATCGA 3’
DNA invader	5’ ATACGTCCTGATAGTTAGTATCCATCGA -FAM 3’
RNA invader	5’ rArUrArCrGrUrCrCrUrGrArUrArGrUrUrArGrUrArUrCrCrArUrCrGrA-FAM 3’
**Replication assays**	
5’ tail strand (upper strand)	5’ TTTTTTTTTTTTTTTTTTTTTTTTTTTTTTTTTTTTTTTTTTTTTAGCAAGCTCGTACCGTGATCATGCGGCAGGTGTAAGCAC -(Invert dT) 3’
3’ tail strand (lower strand)	5’ AGTGCTTACACCTGCCGCATGATCACGGTACGAGCTTGCTTAGGCGAGGTTCGGGACTATCCCTACTTCCAA -(Invert dT) 3’
FAM-labeled DNA primer	5’ FAM-ATTGGAAGTAGGGATAGTCCCGAACCTCGC 3’
40-nt RNA Trap	5’ rArGrCrArArGrCrUrCrGrUrArCrCrGrUrGrArUrCrArUrGrCrGrGrCrArGrGrUrGrUrArArGrCrArCrU 3’
Primer with ddC	5’ ATTGGAAGTAGGGATAGTCCCGAACCTCGC/3ddC/ 3’
20-nt FAM RNA Trap	5’ FAM-rArGrCrArArGrCrUrCrGrUrArCrCrGrUrGrArUrC 3’
Mismatched US	5’ TTTTTTTTTTTTTTTTTTTTTTTTTTTTTTTTTTTTTTTTTTTTTAGCAAGCTCGTACCGTGATCCCGCGGCAGGTGTAAGCAC-(Invert dT) 3’

Supplementary Table S1. Nucleotide sequences of oligonucleotides used in the study.
